# A novel flatworm-specific gene implicated in reproduction in *Macrostomum lignano*

**DOI:** 10.1038/s41598-018-21107-4

**Published:** 2018-02-16

**Authors:** Magda Grudniewska, Stijn Mouton, Margriet Grelling, Anouk H. G. Wolters, Jeroen Kuipers, Ben N. G. Giepmans, Eugene Berezikov

**Affiliations:** 1 European Research Institute for the Biology of Ageing, University of Groningen, University Medical Center Groningen, Antonius Deusinglaan 1, 9713AV Groningen, The Netherlands; 2 Department of Cell Biology, University of Groningen, University Medical Center Groningen, Antonius Deusinglaan 1, 9713AV Groningen, The Netherlands

## Abstract

Free-living flatworms, such as the planarian *Schmidtea mediterranea*, are extensively used as model organisms to study stem cells and regeneration. The majority of flatworm studies so far focused on broadly conserved genes. However, investigating what makes these animals different is equally informative for understanding its biology and might have biomedical value. We re-analyzed the neoblast and germline transcriptional signatures of the flatworm *M*. *lignano* using an improved transcriptome assembly and show that germline-enriched genes have a high fraction of flatworm-specific genes. We further identified the *Mlig-sperm1* gene as a member of a novel gene family conserved only in free-living flatworms and essential for producing healthy spermatozoa. In addition, we established a whole-animal electron microscopy atlas (nanotomy) to visualize the ultrastructure of the testes in wild type worms, but also as a reference platform for different ultrastructural studies in *M*. *lignano*. This work demonstrates that investigation of flatworm-specific genes is crucial for understanding flatworm biology and establishes a basis for such future research in *M*. *lignano*.

## Introduction

Animal models inspired researchers for hundreds of years. In biomedicine, a variety of organisms is employed to study e.g. development, ageing, and mechanistic underpinnings of diseases, with the aim of translating these findings to humans. While most research focusses on the use of established models, such as yeast, nematodes, fruit flies, and mice, it is sometimes the unique feature of a non-standard experimental model that brings the breakthrough. For example, squalamine, a compound isolated from dogfish sharks, exhibits strong anti-fungal and anti-bacterial activity. It was shown to be very efficient in fighting a broad spectrum of human pathogens, strengthening its therapeutic potential^1^. Another example is a recent study deciphering the remarkable resistance of tardigrades to X-ray radiation, which led to the discovery of a novel DNA protector, the Dsup protein. When expressed in human cells, this protein shows the ability to protect human DNA as well^2^. These examples indicate the power of exploring nature’s biodiversity, with a focus on organisms demonstrating extreme characteristics, such as a remarkable resistance to environmental factors, astonishing regeneration abilities, or an extremely long lifespan^3–6^.

**Figure 1:**
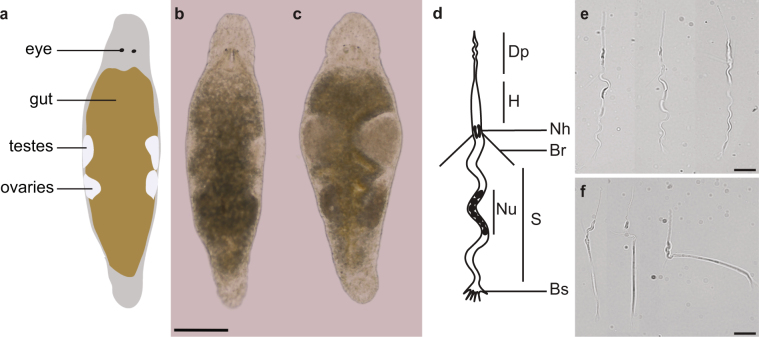
The structure of testes and sperm cells in healthy control and *Mlig-sperm1* knockdown *M*. *lignano* animals. (**a**) Illustration of the simplified morphology of *M*. *lignano*, indicating the location of the eyes, gut, testes, and ovaries. (**b**,**c**) Comparison of the overall morphology between control (**b**) and *Mlig-sperm1(RNAi)* (**c**) individuals. (**d**) Schematic structure of an adult sperm cell. Dp, distal process (feeler); H, head; Nh, notch; Br, bristle; Nu, nucleus; S, shaft; Bs, brush. For a detailed description of sperm cells in *M*. *lignano* see Ref.^16^. (**e**,**f**) Comparison of sperm morphology between control (**e**) and knockdown (**f**) worms using DIC microscopy. Scale bars are 100 µm (**b**,**c**) and 10 µm (**e**,**f**).

One of such organisms is the free-living, hermaphrodite flatworm *Macrostomum lignano* (Fig. 1a), which has a number of interesting features such as the ability of whole-body regeneration^7^, a high resistance to ionizing irradiation up to 210 Gray^8^, and the presence of a population of actively proliferating neoblasts, which represent the stem cells and progenitors of flatworms^9–11^. Recently, we established the transcriptional signatures of the proliferating somatic neoblasts and germline cells, and demonstrated the role of several genes conserved between *M*. *lignano* and human in stem cell and germline biology^12^. To understand the biology of this model organism, it will, however, be crucial to also perform functional studies of non-conserved genes. This is illustrated for example by the recent identification of three novel *Mlig-stylet* genes, which are required for the differentiation of the male copulatory apparatus during tail-regeneration^13^. Importantly, investigating non-conserved genes may also lead to discoveries translatable to human health, such as improved wound healing or novel anthelminthic drugs. The present study demonstrates the groundwork we laid for this novel research direction. As a first step, we reanalyzed the published dataset using an improved transcriptome assembly, and reassessed the conservation level of genes enriched in the neoblasts and germline cells. In addition, we present the characterization of a previously uncharacterized gene which we named *Mlig-sperm1*. Knockdown of this gene results in aberrations in the gonads and sperm structure, and leads to a reduced fertility. This serves as a case study to illustrate the recently developed techniques and resources allowing in depth gene characterization of *M*. *lignano*.

## Results

### Extended *M*. *lignano* transcriptome assembly

As part of the *M*. *lignano* genome annotation we have recently published a genome-guided transcriptome assembly Mlig_RNA_3_7_DV1_v1^14^. We noticed that some real but lowly expressed genes, such as TERT^15^, were not included in this transcriptome assembly because all predicted transcripts with low expression levels (RPKM < 0.5) were filtered out. To mitigate this problem, we extended the transcriptome assembly by keeping low expression transcripts if they contain a predicted open reading frame of at least 100 amino acids. The new transcriptome assembly, Mlig_RNA_3_7_DV1_v3, has 143,648 transcriptional units, which are processed into 153,985 genes after identification of trans-splicing events, and include duplicated gene copies and alternative transcript forms (Supplementary Table 1). Clustering of sequences by 95% global sequence identity results in 53,480 and 88,426 non-redundant transcriptional units and genes respectively (Supplementary Table 1).

### Characterization of conservation levels in different gene groups

To identify the conservation levels of genes enriched in proliferating cells, we first re-analyzed the previously established proliferating neoblast and germline transcriptional signatures^12^ using the extended transcriptome assembly Mlig_RNA_3_7_DV1_v3 (Supplementary Table 2, Supplementary Fig. 1). While the conclusions of the previous work do not change with the new analysis, the number of transcript clusters classified as ‘stringent neoblast’ increased from 357 to 489 (Supplementary Fig. 1k); in contrast, the number of ‘irradiation’ and ‘germline’ transcript clusters decreased from 7,277 to 5,901 and from 2,739 to 2,604 respectively (Supplementary Fig. 1c,g). We assigned the conservation level to each *M*. *lignano* gene as ‘conserved’ if there is a significant homology with human genes, as ‘flatworm-specific’ if homologs are identified only in the free-living *Schmidtea mediterranea* and/or the parasite *Schistosoma mansoni*, or as ‘*M*. *lignano*-specific’ if no homologs are detected (Table 1). The distribution analysis of the conservation levels between different gene categories revealed striking differences between neoblast and germline genes. While overall 47.3% of *M*. *lignano* genes are conserved in human, 8.2% are flatworm-specific and 44.5% are *Macrostomum*-specific (Table 1), for the neoblast genes the fraction of human-conserved genes is substantially higher at 85%, while flatworm-specific and non-conserved gene fractions are only 2.8% and 12.2% respectively (Table 1). At the same time, the fraction of germline genes conserved in human is 37.6%, which is significantly less than overall, while the fraction of *Macrostomum*-specific genes rises to 54.2% (Table 1). Since in this analysis we used all transcripts from the Mlig_RNA_3_7_DV1_v3 transcriptome assembly, including transcripts without predicted open reading frame (ORF), it is possible that the fraction of *Macrostomum*-specific transcripts is inflated. We repeated the conservation distribution analysis using only transcripts with ORFs and clustering sequences at 95% amino-acid identity level to exclude biases due to possible expansions of gene families. However, the picture did not change significantly: overall 55.3% of genes are conserved in human, 9.7% are flatworm-specific and 35% are *Macrostomum*-specific, while the numbers are 86%, 3% and 11% for the neoblast genes and 42.5%, 9% and 48.5% for the germline genes respectively (Table 1).

**Table 1:** Conservation of different *M*. *lignano* gene groups in human and flatworms.

	Total number	Homologs in human	Homologs in *S*. *mediterranea*	Homologs in *S*. *mansoni*	Flatworm- specific*	*M*. *lignano*- specific
**Transcript clusters**						
All	50,673	23,969 (47.30%)	25,716 (50.75%)	21,581 (42.59%)	4,159 (8.21%)	22,545 (44.49%)
Neoblasts	1,062	902 (84.93%)	871 (82.02%)	838 (78.91%)	30 (2.82%)	130 (12.24%)
Neoblasts, stringent	489	406 (83.03%)	379 (77.51%)	370 (75.66%)	14 (2.86%)	69 (14.11%)
Germline	2,604	979 (37.60%)	1067 (40.98%)	840 (32.26%)	213 (8.18%)	1,412 (54.22%)
**Protein clusters**						
All	30,017	16,605 (55.32%)	17,758 (59.16%)	14,819 (49.37%)	2,913 (9.70%)	10,499 (34.98%)
Neoblasts	840	723 (86.07%)	693 (82.50%)	676 (80.48%)	25 (2.98%)	92 (10.95%)
Neoblasts, stringent	404	336 (83.17%)	310 (76.73%)	307 (75.99%)	10 (2.48%)	58 (14.36%)
Germline	1,917	815 (42.51%)	882 (46.01%)	707 (36.88%)	173 (9.02%)	929 (48.46%)

*Present in *S*. *mediterranea* or *S*. *mansoni* but not in human.

### Knockdown of a flatworm-specific gene *Mlig-sperm1* causes abnormal morphology of testes and spermatozoa and decreased fertility

To assess the possible roles of non-conserved and flatworm-specific genes in *Macrostomum* biology, we randomly chose six candidate genes enriched in proliferating cells for a pilot functional screen (Supplementary Table 3). From the tested candidates, one of the germline genes, *Mlig020950*, demonstrated a strong phenotype. We named the *Mlig020950* gene as *Mlig-sperm1* due to severe defects in sperm morphology in *Mlig020950(RNAi)* animals, as described below.

Gene knockdown of *Mlig-sperm1* led to a dramatic enlargement of the testes in all individuals (Fig. 1b,c). Detailed analysis revealed that these oversized testes accumulated large amounts of sperm cells (Fig. 1c–f), characterized by an aberrant morphology (teratozoospermia), such as a very rigid shaft, and often forming contortion at the notch site (Fig. 1f). In contrast to sperm of control animals, which demonstrate undulating movements of the shaft and distal process (Supplementary Video 1), the knockdown worms’ spermatozoa showed atypical motility (asthenozoospermia): cells were not swimming actively and performed twitching movements (Supplementary Video 2).

**Figure 2:**
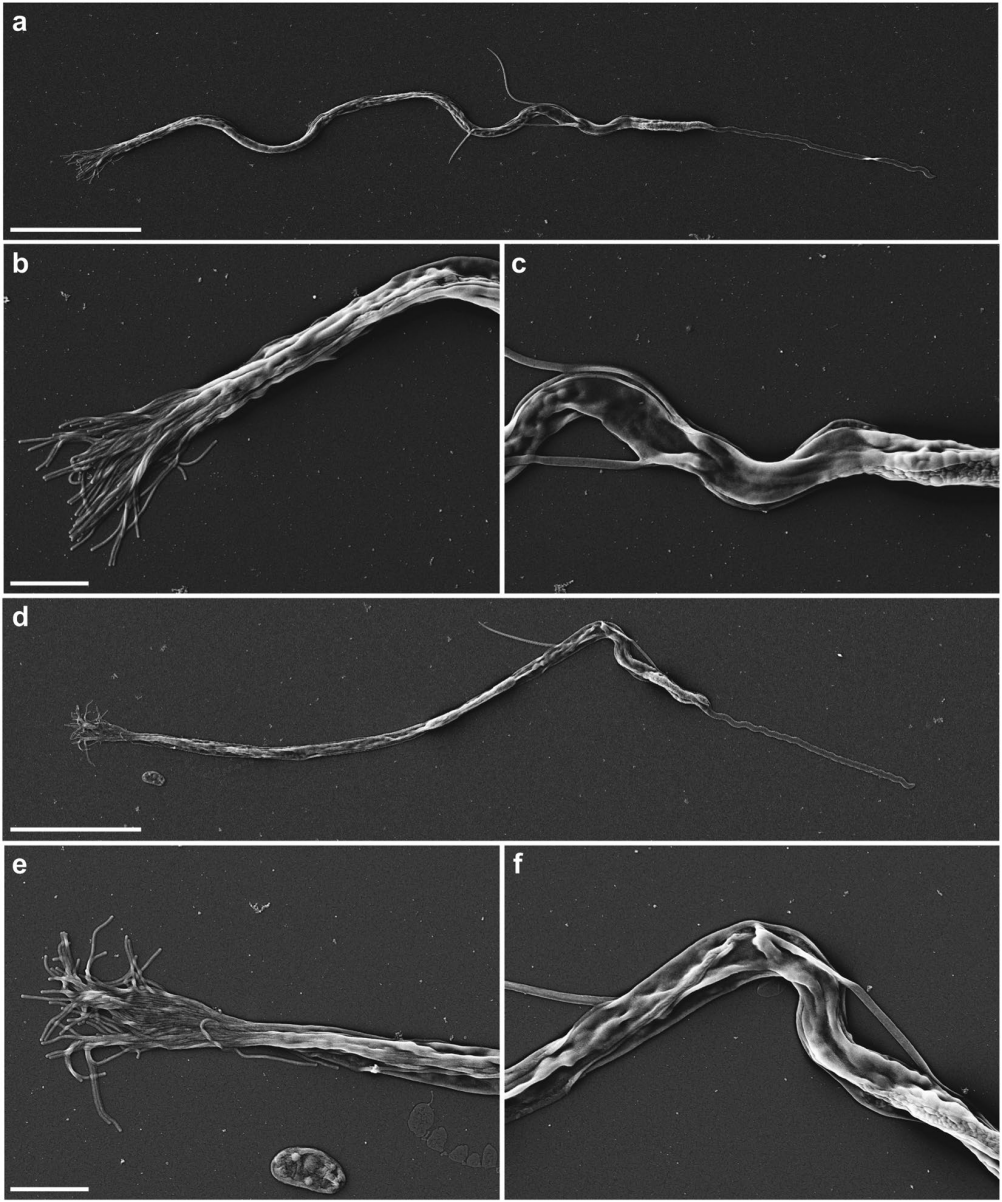
Scanning electron microscopy of spermatozoa. (**a–c**) Spermatozoon of a negative control *GFP(RNAi)* worm. (**a**) Overview of the complete cell. Note the curved view of the shaft. (**b**) Detail of the brush, consisting out of separate extensions. (**c**) Detail of the notch region of the cell. (**d–f**) Spermatozoon of a *Mlig-sperm1(RNAi)* worm. (**d**) Overview of the complete cell. Note the rigidity of the shaft and the contortion at the notch site. (**e**) Detail of the brush. Compared to the negative control the brush looks more flattened with the base of the extensions being more packed together. (**f**) Detail of the notch region clearly showing the contortion. Scale bar are 10 µm (**a**,**d**) and 2 µm (**b**,**c**,**e**,**f**).

**Figure 3:**
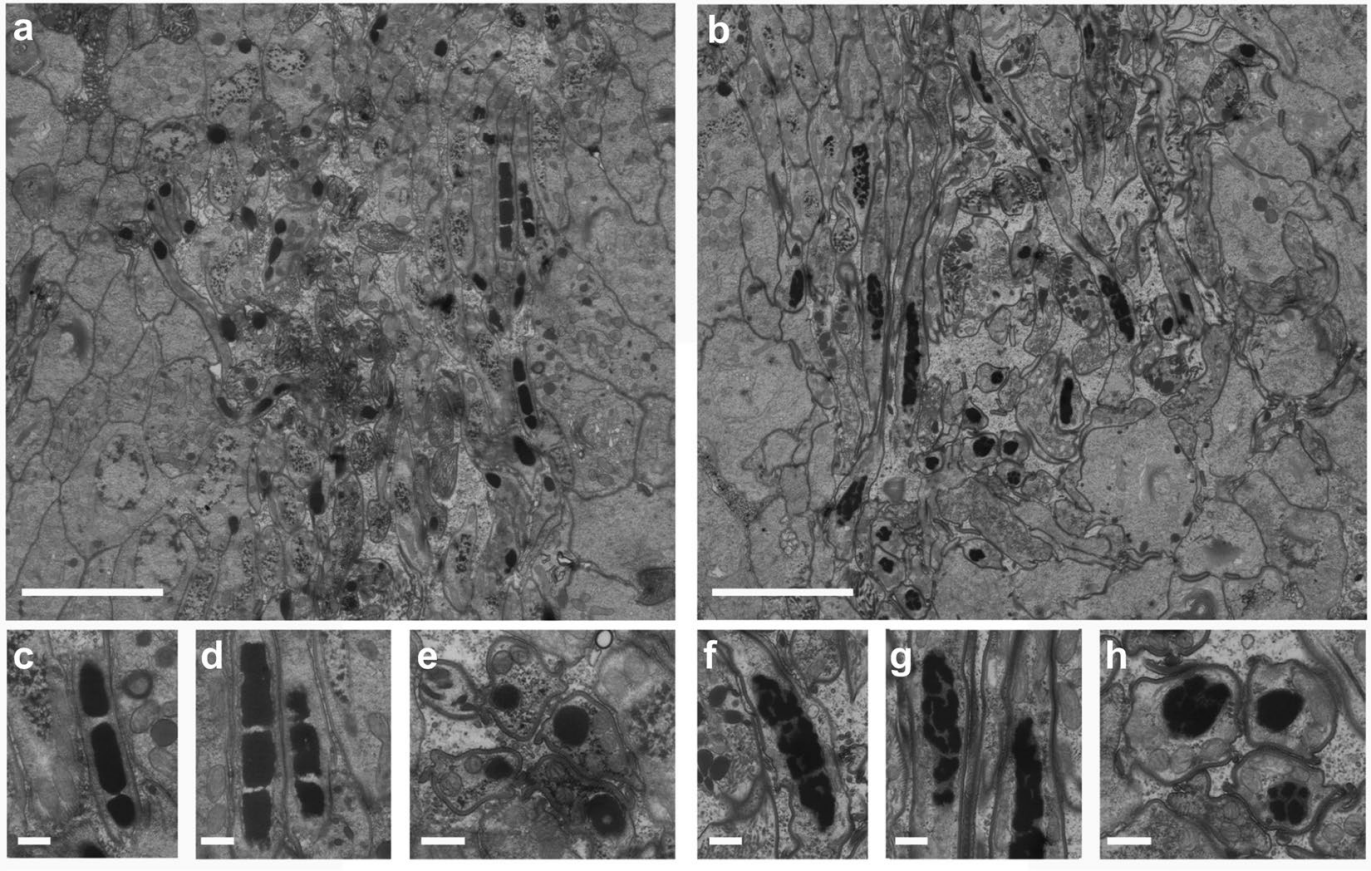
Ultrastructure of spermatids and spermatozoa. (**a**,**b**) Overview of early and late spermatids and spermatozoa in the testes of a negative control *GFP(RNAi)* worm (**a**) and a *Mlig-sperm1(RNAi)* worm (**b**). In both images, several longitudinal and cross sections of nuclei of the spermatozoa can be observed as black structures. (**c**,**d**) Detail of longitudinal sections of spermatozoa nuclei of a *GFP(RNAi)* worm. The chromatin of the nucleus is condensed into discrete bodies. (**e**) Detail of a cross section of spermatozoa nuclei of a *GFP(RNAi)* worm. (**f**,**g**) Detail of longitudinal sections of spermatozoa nuclei of a *Mlig-sperm1(RNAi)* worm. Compared to the negative control, the chromatin of the nuclei looks fragmented and condensation into discrete bodies is less visible. (**h**) Detail of a cross section of spermatozoa nuclei of a *Mlig-sperm1(RNAi)* worm. Compared to the negative control, the chromatin of the nuclei is more fragmented. Scale bars are 5 µm (**a**,**b**) and 500 nm (**c**–**h**).

Scanning electron microscopy (EM) confirmed the morphological aberrancy of *Mlig-sperm1(RNAi)* spermatozoa (Fig. 2). In comparison to control cells (Fig. 2a–c), the shaft of the *Mlig-sperm1* knockdown spermatozoa, demonstrates rigidity and lack of curvature (Fig. 2d), and its brush is flattened and inflexible (Fig. 2e). Furthermore, the contortion at the notch site is clearly visible (Fig. 2f). In addition, transmission EM demonstrated that also the nuclei of late spermatids and spermatozoa of *Mlig-sperm1(RNAi)* worms have an aberrant morphology (Fig. 3). In *GFP(RNAi)* worms, which are used as negative controls, the nucleus elongates and chromatin condenses into a number of discrete bodies connected by small bridges during the late phase of spermiogenesis (Fig. 3a,c,d,e**)** as previously described^16^. In *Mlig-sperm1(RNAi)* worms, distinct nuclear bodies can be rarely observed and the chromatin has a fragmented appearance (Fig. 3b,f,g,h). To enable comparison of the ultrastructure of *GFP(RNAi)* and *Mlig-sperm1(RNAi)* worms with untreated animals, we also provided a nanotomy-atlas of a wild-type individual (Supplementary Fig. 2). In these sections, the fragmented chromatin appearance cannot be observed, indicating that this is indeed a specific characteristic of the phenotype of the *Mlig-sperm1* knockdown.

**Figure 4:**
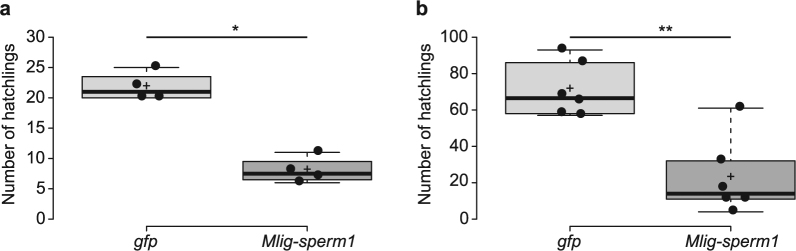
Fertility of healthy control and *Mlig-sperm1* knockdown *M*. *lignano* animals. (**a**) Fertility of 4 groups of *Mlig-sperm1(RNAi)* worms and 4 groups of adult negative control *GFP(RNAi)* worms. The RNAi treatment started with adult animals and was continued for a total of 3 weeks. Data points are indicated by black dots, the black line represents the median, and the cross indicates the mean. The data points represent the number of hatchlings produced by each group of five worms within three weeks. The star represents a significant difference with a p-value < 0.05. (**b**) Fertility of 6 groups of *Mlig-sperm1(RNAi)* worms and 6 groups of negative control *GFP(RNAi)* worms. The RNAi treatment started with one-day old hatchlings and was continued for a total of 5 weeks. The number of hatchlings produced by each group of 5 worms within this period is visualized as a boxplot combined with a beeswarm of the data points. Data points are indicated by black dots, the black line represents the median, and the cross indicates the mean. The double star represents a significant difference with a p-value < 0.01.

As sperm defects are often reported as a cause of decreased male fertility or even complete sterility^17,18^, we compared fertility of control and *Mlig-sperm1(RNAi)* animals. The knockdown of the *Mlig-sperm1* gene in adult animals resulted in a significantly lower (p < 0.05, *Mann-Whitney U test*) number of progeny produced in a period of three weeks (Fig. 4a). To study if knockdown could result in sterility, we performed an additional RNAi experiment of 5 weeks, starting with one-day old hatchlings. In both the control and *Mlig-sperm1(RNAi)* condition, the first juveniles were observed during the second week of treatment. This experiment confirmed the decreased fertility due to *Mlig-sperm1* knockdown (p < 0.01, *Mann-Whitney U test)*, but a complete sterility could not be observed (Fig. 4b).

### *Mlig-sperm1* is expressed exclusively in testes

**Figure 5:**
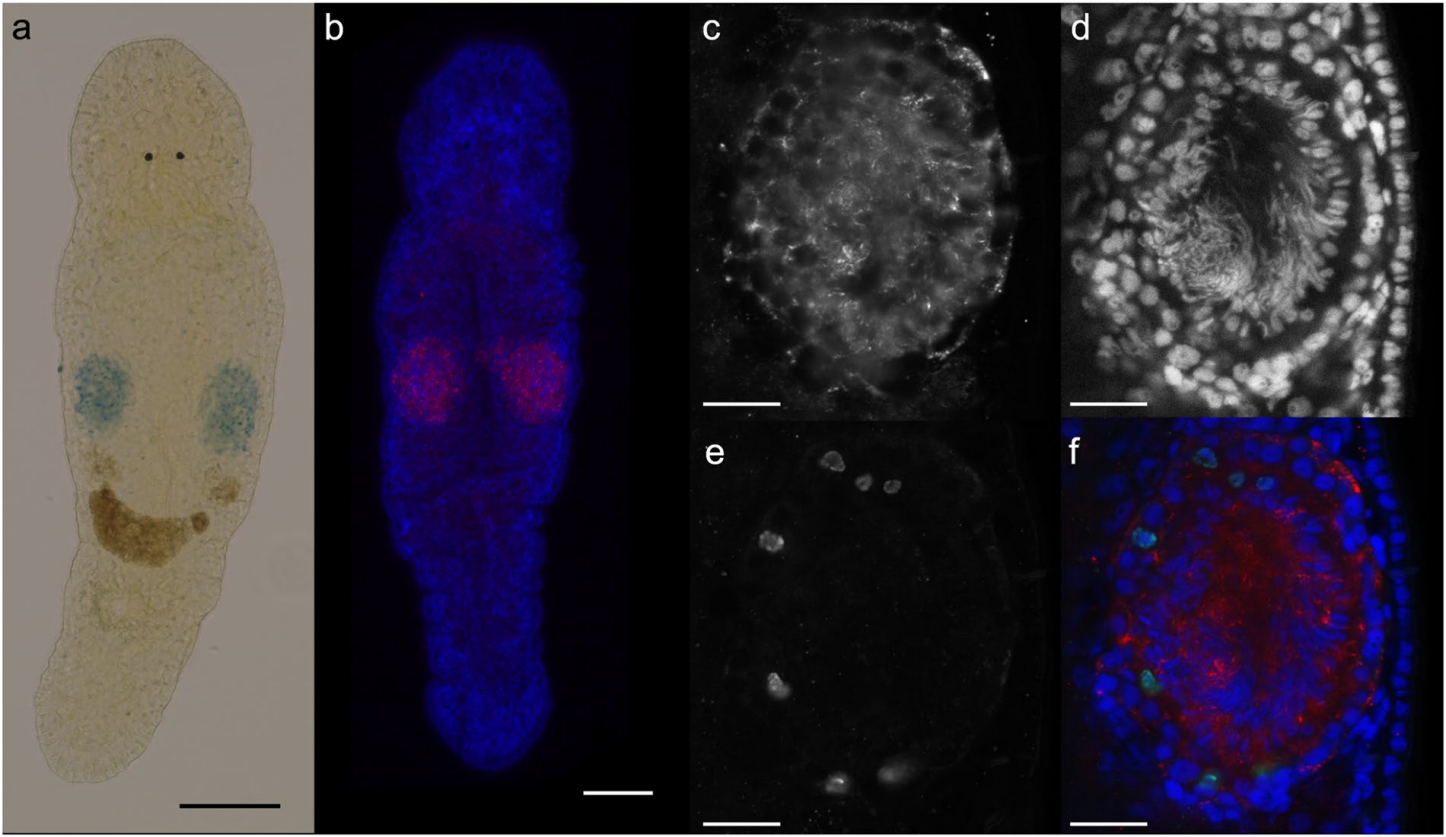
Expression pattern of *Mlig-sperm1* gene. (**a**) Whole mount *in situ* hybridization demonstrating the specific expression of the *Mlig-sperm1* gene in the testes of an adult worm (blue precipitation). (**b**) Fluorescent *in situ* hybridization of the *Mlig-sperm1* gene (red) in an adult worm, combined with a DAPI labeling of all nuclei in the worm (blue). This confirms the testes-specific expression of *Mlig-sperm1*. (**c–f**) Triple labeling including *Mlig-sperm1* FISH (**c**), DAPI labeling (**d**), mitotic labeling with the anti-phospho-histone H3 antibody (**e**), and the overlay of all three (**f**). The *Mlig-sperm1* (red) clearly encircles DAPI-labeled nuclei (blue) indicating the expression within testicular cells. Importantly, this includes the nuclei of the mitotic cells (green), demonstrating expression in the proliferating testicular cells. Scale bars: 100 µm (**a**,**b**), and 25 µm (**c**–**f**).

*Mlig-sperm1* is categorized as enriched in proliferating germline cells according to the RNA-seq data (Supplementary Table 2). To confirm this, and to study the gene expression pattern in more detail, we performed whole mount *in situ* hybridization (WISH). In adult animals, strong expression of *Mlig-sperm1* was observed in the complete testes (Fig. 5a). Fluorescent *in situ* hybridization (FISH) and confocal microscopy analysis of adult worms confirmed the pattern observed with WISH (Fig. 5b). Counterstaining the nuclei of all cells with DAPI indicated that *Mlig-sperm1* is expressed within testicular cells, since the *in situ* pattern encircles the DAPI-labeled nuclei (Fig. 5c,d,f, and Supplementary Fig. 3e–g). In addition, this labeling further indicated that the expression is specific for the testes and is not present in the ovaries (Supplementary Fig. 3g). To further confirm the testicular expression, WISH was performed in one day old hatchlings, and 4–5 days old juveniles. Based on light microscopic analysis, no signal was detected in hatchlings (Supplementary Fig. 3a), suggesting that the gene is not expressed in the gonad anlage of primordial germ cells^11^. In the juveniles, the signal was observed on the level of developing testes in a cluster of several cells (Supplementary Fig. 3b). The expression of *Mlig-sperm1* in proliferating testicular cells was confirmed by a FISH combined with a DAPI and mitotic counterstain. Figure 5(c–f) demonstrates *Mlig-sperm1* expression around the labeled mitotic nuclei. In conclusion, *Mlig-sperm1* is expressed in both proliferating testicular cells and non-proliferating cells of the testes such as spermatids and spermatozoa. Specificity of ISH labelling was confirmed by a *Mlig-sperm1* sense probe, for which no signal was detected (Supplementary Fig. 3d).

### Mlig-sperm1 is a member of a large gene family specific for free-living flatworms

The *Mlig-sperm1* gene has two nearly identical loci in the Mlig_3_7_DV1 genome assembly, *Mlig020950*.*g1* and *Mlig020950*.g2. We next studied the relation of this gene to other *Macrostomum* genes and homologs in other flatworms. The BLAST search revealed that *Mlig-sperm1* has a significant similarity with 35 genes in *M*. *lignano* (blastp e-value cutoff 1e-15), which can be grouped into 14 protein clusters based on 95% amino-acid identity cutoff (Supplementary Table 4). Notably, most of the homolog genes are enriched in the proliferating germline cells according to the previous analysis^12^. Furthermore, the gene is conserved in *S*. *mediterranea*, with one and eight contigs identified in asexual (dd_Smed_v6) and sexual (dd_Smes_v1) transcriptome assemblies respectively (Supplementary Table 4), as well as in all 5 other planarian species available in PlanMine^19^. We did not find significant matches to the Mlig-SPERM1 protein in the transcriptomes of parasitic flatworms or outside flatworm species. Alignment of the representative proteins revealed a conserved domain common to all genes (Supplementary Fig. 4). However, a search against the Pfam database did not reveal homology to any known protein family. The tertiary structure of the Mlig-SPERM1 protein, as predicted by RaptorX^20^, consists of three domains with 48% of the predicted positions being disorganized. The region conserved between all the genes (Supplementary Fig. 4) corresponds to the first domain, which has the highest organization factor and consists of one beta sheet and several alpha helixes (Supplementary Fig. 5).

## Discussion

To investigate the extent of conservation of *M*. *lignano* genes with neoblast- and germline-enriched expression, we first reanalyzed the transcriptomic signatures of proliferating cells in *M*. *lignano*. While all previous RNA-seq based gene expression studies in *M*. *lignano* relied on *de novo* transcriptome assemblies^12,13,21,22^, we here used the genome-guided transcriptome assembly Mlig_RNA_3_7_DV1_v3, which is based on the recently generated and significantly improved genome assembly for the *M*. *lignano* DV1 line^14^. The new transcriptome assembly resolved many partial transcript issues inherent for *de novo* transcriptomes, and the decreased fragmentation allows more accurate estimation of gene conservation depth. This explains the change in numbers of transcript clusters of gene groups as ‘stringent neoblast’, ‘irradiation’, and ‘germline’ when the current analysis is compared to the initial one^12^.

Interestingly, neoblast-enriched genes were found to be deeply conserved, while germline-enriched genes have many non-conserved or flatworm-specific genes. This is in line with published literature on deep conservation of the neoblast regulation program^23^ and significant variation in evolution of reproductive systems^24^.

As a next step, we randomly selected six non-conserved candidate genes for a preliminary functional screen, and demonstrated that one of them, *Mlig-sperm1*, is expressed in all testicular cell types, including proliferating and differentiated cells. Indeed, it should be kept in mind that the enrichment in proliferating germline cells does not give information about the expression in differentiated testicular cell types, a group of cells for which specific transcriptomic analysis is not yet available. All results taken together demonstrate that *Mlig-sperm1* plays a role in forming healthy sperm, and consequently the reproductive capacity of *M*. *lignano*. Knock down of *Mlig-sperm1* resulted in an accumulation of aberrant spermatozoa, leading to enlarged testes. Enlargement of the testes due to accumulating aberrant testicular cells was previously observed during knock down of *melav2*, a gene essential for spermatid differentiation^25^. Besides its role in sperm differentiation, *Mlig-sperm1* might have additional functions, as is it also expressed in the proliferating testicular cells. Given the conservation level between *Mlig-sperm1* and other genes, and the classification of many of the genes as enriched in proliferating germline cells, we suggest that *Mlig-sperm1* is a member of a novel protein family specific for free-living flatworms, with important roles in reproduction.

Of note, the transmission electron microscopy analysis of *GFP(RNAi)* and *Mlig-sperm1(RNAi)* animals presented in Fig. 3 was performed using the anatomy at the nanoscale (nanotomy) approach, which allows visualization of large specimen areas^26^ and provides a ‘Google-Earth’ style of data presentation and navigation at different levels of resolution. The nanotomy datasets are available at http://www.nanotomy.org/OA/Macrostomum. In addition to the RNAi worms, the ultrastructure of wild-type testes is also made available. However, instead of focusing on the testes area, we generated 35 cross-sections and a longitudinal section covering a complete animal (Supplementary Fig. 2). While the detailed annotation of these nanotomy images is beyond the scope of this work, we believe that the generated resource will serve as a valuable reference on *M*. *lignano* morphology at the ultrastructural level and complements genomic resources available for this developing model organism. The current progress in the available resources and techniques, e.g. the recently developed transgenesis methods^14^, are making this model increasingly attractive for different research areas.

Our approach of specifically selecting non-conserved or flatworm-specific genes can yield important insight into aspects of flatworm biology, such as germline development. This is illustrated by the *Mlig-sperm1*, but also the recently published example of *Mlig-stylet* genes^13^. Future studies of genes conserved within flatworms, and more specifically in parasitic flatworms, could help to develop treatments for infections caused by parasites, such as *Schistosoma*. Current research in that area focuses its efforts on dissecting the mechanism behind the maintenance and activity of the germline, as the egg-induced inflammation is the main cause of *Schistosoma*-associated pathologies^27,28^. In addition, focusing on somatic neoblast-enriched flatworm-specific genes could contribute to our understanding of e.g. their astonishing regeneration capacity and would create an opportunity to improve such competencies in humans. This is in line with the recent report on improved radiotolerance of human cultured cells by a tardigrade-unique protein^2^. We therefore advocate that investigating *M*. *lignano* genes not conserved in humans is an approach with truly great potential. This paper illustrates several of the current available tools and resources for this type of research.

## Methods

### Experimental organism and culture conditions

*Macrostomum lignano* (Macrostomida, Rhabditophora) is a free-living marine flatworm and an obligatory, non-self-fertilizing hermaphrodite reproducing exclusively in a sexual manner^29^. The combination of a short generation time of about 3 weeks and high fertility rates allows a rapid expansion of cultures^30^.

The animal is small, about 1 mm long and consists of approximately 25,000 cells^9^. Worms are transparent and major tissues and organs can be easily distinguished (i.e. eyes, brain, gonads, reproductive organs, gut). Worms are kept in Petri dishes with nutrient-enriched artificial seawater (f/2)^31^, at 20 °C and a 14/10 hours light/dark cycle and are fed *ad libitum* with the diatom *Nitzschia curvilineata*^29^.

In the present study, the recently collected and introduced in the Berezikov lab, NL10 strain was used. In contrast to DV1^32^, NL10 does not demonstrate chromosomal polymorphism^14^.

### Transcriptome assembly

The transcriptome assembly Mlig_RNA_3_7_v3 was generated exactly as previously described, with the exception that low expressed transcripts (RPKM < 0.5) were retained in the assembly if they contain on open reading frame of at least 100 amino-acids, as predicted by as predicted by TransDecoder^33^. Furthermore, the transcripts in the assembly were classified by selecting only one representative transcript for each predicted CDS and removing the non-coding transcripts and transcripts overlapping repeat annotations. The resulting transcriptome subset is named ‘Core Genes’ and contains 56,036 non-redundant genes (Supplementary Table 1). This Core Genes subset of the Mlig_RNA_3_7_DV1_v3 transcriptome assembly is useful in cases where representative transcripts rather than full transcriptome diversity is desirable.

### Conservation and alignments

Amino-acid sequences from the Mlig_RNA_3_7_DV1_v3 transcriptome assembly were queried against human, *S*. *mediterranea* (dd_Smed_v6 and dd_Smes_v1) and *S*. *mansoni* (ASM23792v2) genes using BLAST^34^ and hits with E-value below 0.01 were considered as homologs for the purpose of conservation analysis. In addition, *Mlig-sperm1* gene was search against all species available in PlanMine^19^ and WormBase^35^. Sequence alignment and visualization were performed with CLC Main Workbench (QIAGEN Aarhus A/S).

### Whole mount *in situ* hybridization

cDNA synthesis was performed using the SuperScript III First-Strand Synthesis System (Life Technologies) according to the manufacturer’s protocol with 2–3 µg of total RNA as a template for each reaction. Provided oligo(dT) and hexamer random primers were used.

DNA fragments selected as templates for *in situ* hybridization probes, were amplified from cDNA by standard PCR with GoTaq Flexi DNA Polymerase (Promega), followed by cloning using the pGEM-T vector system (Promega) and sequenced by GATC Biotech. All primers used are listed in Supplementary table 3. DNA templates for producing DIG – labeled riboprobes were amplified from sequenced plasmids using High Fidelity Pfu polymerase (Thermo Scientific). Forward (5′-CGGCCGCCATGGCCGCGGGA-3′) and reversed (5′TGCAGGCGGCCGCACTAGTG-3′) primers binding the pGEM-T vector backbone near the insertion site were designed. Moreover, versions of the same primers with a T7 promoter sequence (5′-GGATCCTAATACGACTCACTATAGG-3′) appended upstream were obtained. The T7 promoter sequence serves as a start site in subsequent *in vitro* transcriptions. A pair of primers, depending on the orientation of the insert in the vector: forward with T7 promoter and reverse without or vice versa, was used to amplify every ISH probe template.

Digoxigenin (DIG) labeled RNA probes (800 to 1200 bp in length) were generated using the DIG RNA labeling Mix (Roche, Switzerland) and T7 RNA polymerase (Promega, Fitchburg, WI) according to the manufacturer’s protocol for *in vitro* transcription. The concentration of every probe was measured with the Qubit RNA BR assay (Invitrogen), probes were diluted in Hybridization Mix^10^ to 20 ng/µl, stored at −80 °C and used within 4 months. The final concentration of the probe and optimal temperature used for hybridization varied for different probes and were optimized for each probe.

Whole mount *in situ* hybridization (ISH) was performed following the published protocol^10^. Pictures were made using a standard light microscope with DIC optics and an AxioCam HRC (Zeiss, Germany) digital camera and the EVOS XL Core Imaging System (ThermoFisher).

### Fluorescent *in situ* hybridization and immunofluorescence

Fluorescent *in situ* hybridization (FISH) was performed following the published FastBlue protocol developed for planarians^36^, except the 5% NAC treatment and bleaching steps were omitted. FISH combined with immunofluorescence of mitotic cells was performed as described before^12^ using the primary anti-phopho histone H3 (1:250) (Millipore, Billerica, MA) and secondary goat anti-rabbit IgF Antibody conjugated with FITC (1:150) (Millipore). The DAPI counterstain was always performed as a last step by incubating labeled worms in NucBlue Fixed Cell ReadyProbes Reagent (ThermoFisher Scientific) for at least 1 hour to ensure penetration in the complete worm. Slides were mounted using 80% glycerol solution, and the labeling was visualized with a Leica TCS SP8 confocal microscope at the UMCG Imaging and Microscopy Center.

### RNA interference

In order to generate dsRNA fragments, the same plasmids were used as for making ISH probes. Templates for the synthesis of both sense and antisense RNA strands were amplified from the plasmids containing the insert of interest. The same primers were used as for ISH riboprobe template amplification, and for each fragment, two PCRs were performed – with both pairs of primers (forward with T7 promoter and reversed without and vice versa). High Fidelity Pfu polymerase (Thermo Scientific) in 150 µl of total volume reaction was used. PCR products were then run on 1% agarose gel, PCR product bands were cut out and purified using the QIAquick Gel Extraction Kit (Qiagen, Netherlands). Each template was then used to synthesize the corresponding single strand RNA with the TranscriptAid T7 High Yield Transcription Kit (Thermo Scientific) according to manufacturer’s protocol. The single reaction volume was 50 µl, and tubes were incubated in 37 °C for 5 hours. Afterwards 100 µl of nuclease-free water was added to each tube, sense and antisense RNA strands were mixed to a final volume of 300 µl and annealed by incubating them at 70 °C for 10 min, followed by gradual cooling down to room temperature, taking approximately 90 min. Every sample was then treated with 1U of RNase A (Life Technologies) and 5U of DNase I (Thermo Scientific) for 45 min at 37 °C. Samples were alcohol precipitated overnight at −80 °C. dsRNA was pelleted by centrifugation at 12,000 g for 15 min at 4 °C, washed with 75% ethanol, and air-dried for 5 min. dsRNA was resuspended in nuclease-free water and the concentration was measured using Nanodrop ND1000. Freshly autoclaved and filtered f/2 medium was used to adjust the concentration to 10 ng/µl. Samples were aliquoted in 1.5 ml Eppendorf tubes and stored at −80 °C.

Specific knockdown of candidate genes by RNA interference with double-stranded RNA delivered by soaking was performed as previously described^37^. RNAi soaking experiments were performed in 24-well plates in which algae were grown. Individual wells contained 300 µl of dsRNA solution (10 ng/ml in f/2 medium) in which 15 individuals were maintained. RNAi was performed for three weeks during which dsRNA solution was refreshed daily. Worms were weekly transferred to fresh 24-well plates to ensure sufficient amount of food. As a negative control, GFP dsRNA was used. Photos of randomly selected worms were made between 2 and 3 weeks of treatment.

### Fertility experiment

During the RNAi experiment with adults, worms were treated for three weeks with *Mlig020950* dsRNA or with *GFP* dsRNA as a negative control. After that, worms were randomly selected and divided into four groups of five worms. These were cultured in freshly prepared 12-well plates for three weeks while RNAi treatment was ongoing. As a measure of fertility, the number of hatchlings produced by each group were counted twice a week.

During the RNAi experiment starting during development, six groups of five randomly selected one-day old hatchlings were made. These groups were cultured in freshly prepared 12-well plates for a total of five weeks, including the developmental time (2–3 weeks) and early adulthood. During this time worms were treated with *Mlig020950* dsRNA or with *GFP* dsRNA as a negative control. As a measure of fertility, the number of hatchlings produced by each group within these five weeks were counted on a daily basis.

### Electron Microscopy (EM)

#### Scanning EM to define surface structure using secondary electron detection

To isolate spermatozoa, *Mlig020950(RNAi)* and *GFP(RNAi)* worms were relaxed in 7.14% MgCl.6H_2_O and cut through the testes on a glass slide, using a surgical blade. The cells within the testes were then squeezed out and pipetted onto a poly-l-lysine coated coverslip. After fixation in 2% glutaraldehyde plus 2% paraformaldehyde in 0.1 M sodium cacodylate, samples were postfixed with 1% Osmium tetroxide for 30 minutes at 4 °C. Slides were rinsed three times with water, and dehydrated through increasing concentrations of ethanol. Samples were incubated for 10 min in a 1:1 mixture of absolute ethanol and tetramethylsilane on ice, followed by 10 minutes incubation in pure tetramethylsilane on ice. Samples were air dried, glued on aluminium stubs using double sided carbon tape, sputter coated with 10 nm Pd/Au and imaged in a Zeiss Supra55 Scanning Electron Microscope operated at 5 KV using secondary electron detection (Fig. 2).

#### Transmission EM for ultrastructural analysis of spermatogenesis

*Mlig020950(RNAi)* and *GFP(RNAi)* worms were relaxed in 7.14% MgCl.6H_2_O and fixed in 2% glutaraldehyde plus 2% paraformaldehyde in 0.1 M sodium cacodylate buffer for 24 hours at 4 °C. After postfixation in 1% osmium tetroxide/1.5% potassium ferrocyanide for 2 hours at 4 °C, worms were dehydrated using ethanol and embedded in EPON epoxy resin. Sections of 60 nm were collected on single slot grids and contrasted using 5% uranyl acetate in water for 20 min, followed by Reynolds lead citrate for 2 min. The longitudinal sections were scanned as described before^38^ (Fig. 3).

#### Transmission EM for transversal nanotomy of *Macrostomum* (back scatter detector)

*M*. *lignano* was fixed following the recommended chemical fixation method described by Salvenmoser^39^, using a simultaneous fixation with glutaraldehyde and osmium tetroxide. Worms were dehydrated using ethanol and embedded in EPON epoxy resin. Thin sections (~100 nm) were collected every 30 µm on silicon wafers as described before^40^. Data was acquired on a Zeiss Supra 55 STEM microscope using a back scatter detector (BSD) at 5 kV with 5 nanometer pixel size, 5 µs dwell time using an external scan generator ATLAS 5 (Fibics, Canada) and stitched as described before^38,41^. After tile stitching the data were exported as an html file and uploaded to the online image database (Supplementary Fig. 2). Data are available at http://www.nanotomy.org/OA/Macrostomum. Supplementary Fig. 2.

### Data availability

The extended transcriptome assembly Mlig_RNA_3_7_DV1_v3 is available at http://gb.macgenome.org/downloads. The re-analyzed gene expression data are available at http://neoblast.macgenome.org. The generated nanotomy images are available at http://www.nanotomy.org/OA/Macrostomum.

## Electronic supplementary material

Supplementary materials

Supplementary Table 2

Supplementary video 1

Supplementary video 2
