# Supplementary material for: A novel flatworm-specific gene implicated in reproduction in Macrostomum lignano
Source: Sci Rep. 2018 Feb 16;8:3192. doi: 10.1038/s41598-018-21107-4 (PMC5816591; doi:10.1038/s41598-018-21107-4)
Supplement: Supplementary file 1 — Supplementary materials [file 41598_2018_21107_MOESM1_ESM.pdf]

A novel flatworm-specific gene implicated in reproduction in *Macrostomum lignano*

Magda Grudniewska, Stijn Mouton, Margriet Grelling, Anouk H. G. Wolters, Jeroen Kuipers, Ben N. G. Giepmans, Eugene Berezikov

Supplementary materials

Supplementary Table 1. Characteristics of Mlig_RNA_3_7_DV1_v3 transcriptome assembly.

	Transcriptional Units	Genes	Core Genes
Number of transcripts	143,648	153,985	90,195
Total length	592 Mb	474 Mb	236 Mb
Number of non-redundant sequences[a]	53,480	88,426	56,036
Total length of non-redundant sequences[a]	222 Mb	312 Mb	171 Mb
Average transcript length	4.1 kb	3.1 kb	2.6 kb
Longest transcript	59,238 nt	53,263 nt	49,790 nt
Transcripts with single trans-splicing site	29,559 (20.58%)	51, 241 (33.28%)	27,078 (30.02%)
Transcripts with multiple trans-splicing sites	14,334 (9.98%)	-	-
Transcripts with defined poly(A) site	102,301 (71.22%)	110,737 (71.91%)	60,427 (67.00%)
TransRate score	0.2386	0.2592	0.3154
Average gene length	10.2 kb	7.9 kb	5.5 kb
Average number of introns per gene	5.3	4.8	4.0
Average intron length	1.4 kb	1.3 kb	1.1 kb
Human homolog genes	-	8,913	8,543
PFAM domains	-	6,201	5,975
Eukaryotic BUSCOs (n=303)			
complete	-	298 (98.35%)	298 (98.35%)
fragmented	-	2 (0.66%)	2 (0.66%)
Missing	-	3 (0.99%)	3 (0.99%)

[a] Sequences with >=95% identity at nucleotide level.

Supplementary Table 2. Gene counts, fold changes and FDR for various gene expression comparisons, and classification of genes into categories. Provided as a separate Excel file.

Supplementary Table 3. Genes selected for the RNAi screen.

Gene	Forward primer	Reversed primer	Product size
Mlig005580.g1	CTCATTGTGGTGCTGGAGTC	AAGCGGGAACCTAGGGAATA	1049
Mlig018764.g1	CCTTCATAGGAAGCGTGAGC	CATGTGACACTGCCTGACAA	1071
Mlig015516.g1	GGAGTAGGGCGGCATTTACT	TTGATTGCTGCTTTTTGTCG	1350
Mlig025071.g1	ATAAGGTGGCCATCATCAGC	GAATTCGATTTGCGATCCAT	1020
Mlig020950.g1, Mlig020950.g2	TAGCCCTGCTAAGGCAGGTA	CGATGTTGGCGTTTCTTCTT	1099
Mlig010408.g1, Mlig010408.g2	GTCAGTCATCTCGGCATTCA	CAGGACGTCGTTGCTATGAG	1252

Supplementary Table 4. Members of the *Mlig-sperm1* gene family.

Gene	% Identity	Aln. len.	E-value	Gene group	Transcript Cluster	Protein Cluster	Conserv.
**Mlig020950.g1**	100.00	635	0.0	Germline	MligTCv3c-5901.0	**MligPCv3c-11292**	flatworms
Mlig020950.g2	99.37	635	0.0	Germline	MligTCv3c-5901.0	MligPCv3c-11292	flatworms
**Mlig009246.g1**	79.64	280	4e-173	Germline	MligTCv3c-19851.0	**MligPCv3c-28017**	flatworms
Mlig009246.g3	79.64	280	4e-173	Germline	MligTCv3c-19851.0	MligPCv3c-28017	flatworms
Mlig009246.g2	79.64	280	5e-173	Germline	MligTCv3c-19851.0	MligPCv3c-28017	flatworms
**Mlig012325.g2**	55.97	243	5e-97	Irradiation	MligTCv3c-5898.1	**MligPCv3c-11289**	flatworms
Mlig012325.g4	55.97	243	6e-97	Germline	MligTCv3c-5898.2	MligPCv3c-11289	flatworms
Mlig012325.g3	55.97	243	6e-97	Germline	MligTCv3c-5898.2	MligPCv3c-11289	flatworms
**Mlig031710.g1**	51.22	246	9e-88	Germline	MligTCv3c-18949.1	**MligPCv3c-26913**	flatworms
Mlig017805.g1	51.22	246	1e-87	Germline	MligTCv3c-18949.0	MligPCv3c-26913	flatworms
Mlig019621.g1	51.22	246	6e-87	Irradiation	MligTCv3c-18949.2	MligPCv3c-26913	flatworms
Mlig012325.g1	63.06	157	1e-71		MligTCv3c-5898.0	MligPCv3c-11288	flatworms
**Mlig029294.g2**	37.07	259	8e-53	Germline	MligTCv3c-10161.0	**MligPCv3c-16206**	flatworms
Mlig029294.g1	37.07	259	8e-53	Germline	MligTCv3c-10161.3	MligPCv3c-16206	flatworms
Mlig013082.g1	37.07	259	2e-52	Germline	MligTCv3c-10161.1	MligPCv3c-16206	flatworms
**Mlig019406.g3**	39.75	239	3e-51	Germline	MligTCv3c-20308.0	**MligPCv3c-28574**	flatworms
Mlig019406.g1	39.75	239	3e-51	Germline	MligTCv3c-20308.0	MligPCv3c-28574	flatworms
Mlig019406.g2	39.75	239	3e-51	Germline	MligTCv3c-20308.3	MligPCv3c-28574	flatworms
Mlig001515.g6	40.17	239	9e-50	Germline	MligTCv3c-20308.1	MligPCv3c-28575	flatworms
Mlig001515.g2	40.17	239	1e-49	Germline	MligTCv3c-20308.1	MligPCv3c-28575	flatworms
**Mlig000837.g18**	38.89	162	1e-34	Irradiation	MligTCv3c-19549.0	**MligPCv3c-27670**	flatworms
**Mlig019468.g4**	36.98	192	3e-34	Germline	MligTCv3c-5534.0	**MligPCv3c-10878**	flatworms
Mlig019468.g2	36.98	192	3e-34	Germline	MligTCv3c-5534.0	MligPCv3c-10878	flatworms
Mlig019468.g1	36.98	192	3e-34	Germline	MligTCv3c-5534.0	MligPCv3c-10878	flatworms
Mlig019468.g5	36.98	192	3e-34	Germline	MligTCv3c-5534.1	MligPCv3c-10878	flatworms
Mlig000837.g12	38.89	162	5e-34	Irradiation	MligTCv3c-19549.0	**MligPCv3c-27670**	flatworms
Mlig000837.g5	38.89	162	6e-34	Irradiation	MligTCv3c-19549.0	MligPCv3c-27670	flatworms
**Mlig023921.g1**	33.92	171	6e-27	Irradiation	MligTCv3c-10246.1	**MligPCv3c-16312**	flatworms
**Mlig018401.g2**	34.48	116	5e-17	Germline	MligTCv3c-9825.0	**MligPCv3c-15783**	flatworms
Mlig004316.g2	34.48	116	5e-17	Germline	MligTCv3c-9825.0	MligPCv3c-15783	flatworms
Mlig004316.g1	34.48	116	5e-17	Germline	MligTCv3c-9825.0	MligPCv3c-15783	flatworms
Mlig018401.g3	36.44	118	1e-16	Germline	MligTCv3c-9825.0	MligPCv3c-15783	flatworms
**Mlig034150.g2**	30.37	135	2e-15	Germline	MligTCv3c-14983.1	**MligPCv3c-21986**	flatworms
Mlig034150.g1	30.37	135	2e-15	Germline	MligTCv3c-14983.1	MligPCv3c-21986	flatworms
Mlig034150.g3	30.37	135	3e-15	Germline	MligTCv3c-14983.0	MligPCv3c-21986	flatworms
**dd_Smes_v1_55033_1_1**	34.97	183	2e-31				
dd_Smes_v1_40403_1_1	37.93	145	3e-26				
dd_Smes_v1_1158_1_2	33.80	142	2e-19				
dd_Smes_v1_1158_1_1	33.80	142	2e-19				
dd_Smes_v1_23757_1_1	26.44	174	6e-18				
dd_Smes_v1_35536_1_1	32.37	173	4e-17				
**dd_Smed_v6_22487_0_1**	30.59	170	4e-17				
dd_Smes_v1_11298_1_1	30.59	181	4e-17				
dd_Smes_v1_43688_1_1	30.59	170	3e-17				

* Genes in bold were used in constructing sequence alignment.

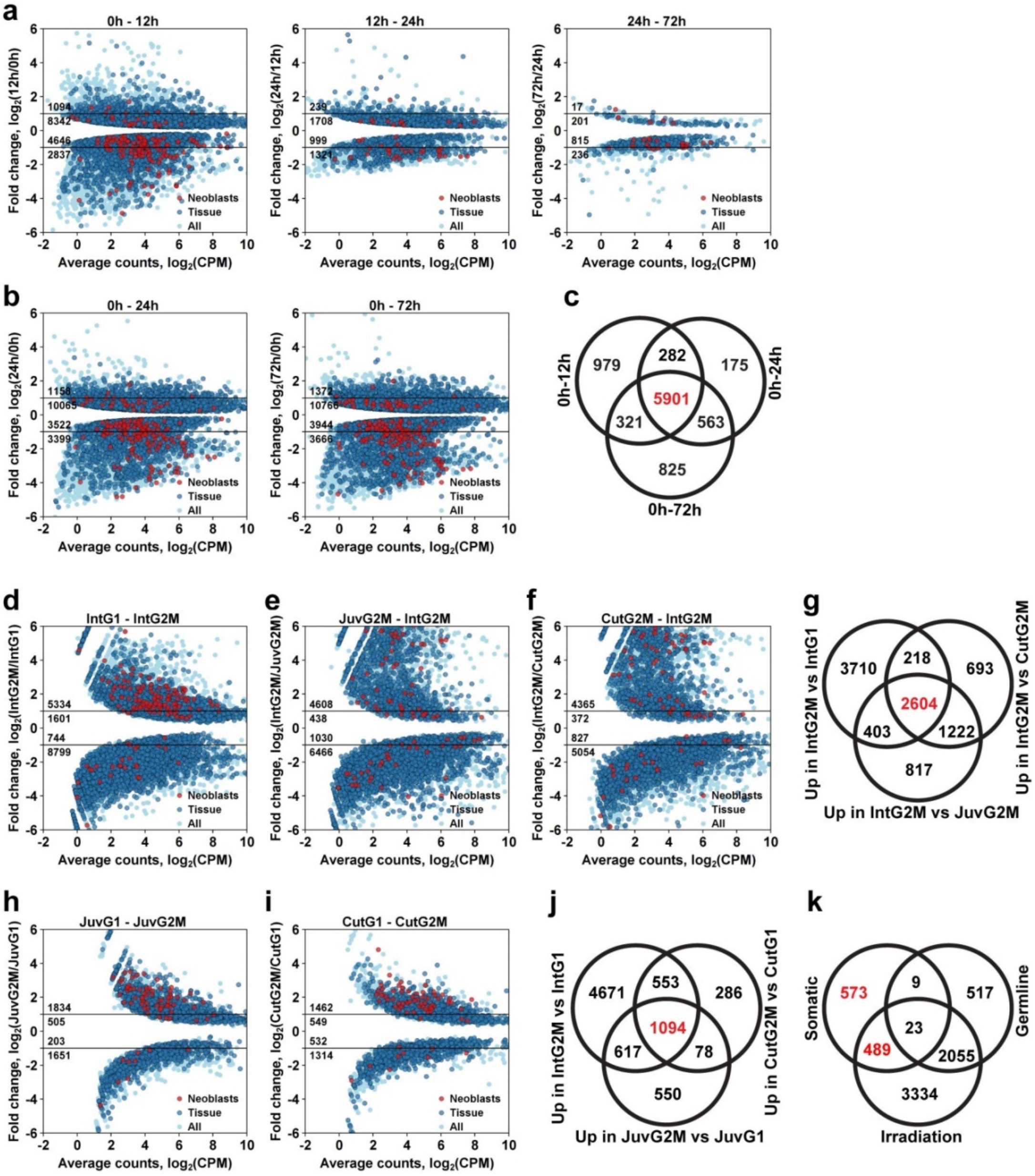

Supplementary Figure 1. Reanalysis of RNA-seq data from Grudniewska et al. (2016) using Mlig_RNA_3_7_DV1_v3 transcriptome assembly. (**a - c**) Identification of differentially expressed genes based on γ-irradiation approach. (**a**) Temporal profile of differentially expressed genes between all three time points. (**b**) Genes differentially expressed between 0h and 24h, and 0h and 72h. (**c**) Venn diagram representation of the number of genes enriched in proliferating cells (indicated in red). (**d - k**) Identification of differentially expressed genes based on FACS approach. (**d**) Genes differentially expressed between differentiated (G1 phase of cell cycle, 2C DNA content) and proliferating (G2/M phase of cell cycle, 4C DNA content) cells of intact worms. (**e**) Genes differentially expressed between proliferating cells of juvenile and intact worms. (**f**) Genes differentially expressed between proliferating cells of cut and intact worms. (**g**) Venn diagram representation of the number of genes enriched in the germline (indicated in red). (**h**) Genes differentially expressed between differentiated and proliferating cells of juvenile worms. (**i**) Genes differentially expressed between differentiated and proliferating cells of cut worms. (**j**) Venn diagram representation of the number of genes enriched in somatic neoblasts (indicated in red). (**k**) Venn diagram representation of the number of genes enriched in somatic neoblasts based on both approaches: irradiation and FACS (indicated in red). Classification of genes as 'Neoblast' and 'Tissue' is based on homology to S. mediterranea genes from Wurtzel et al. (2015).

a

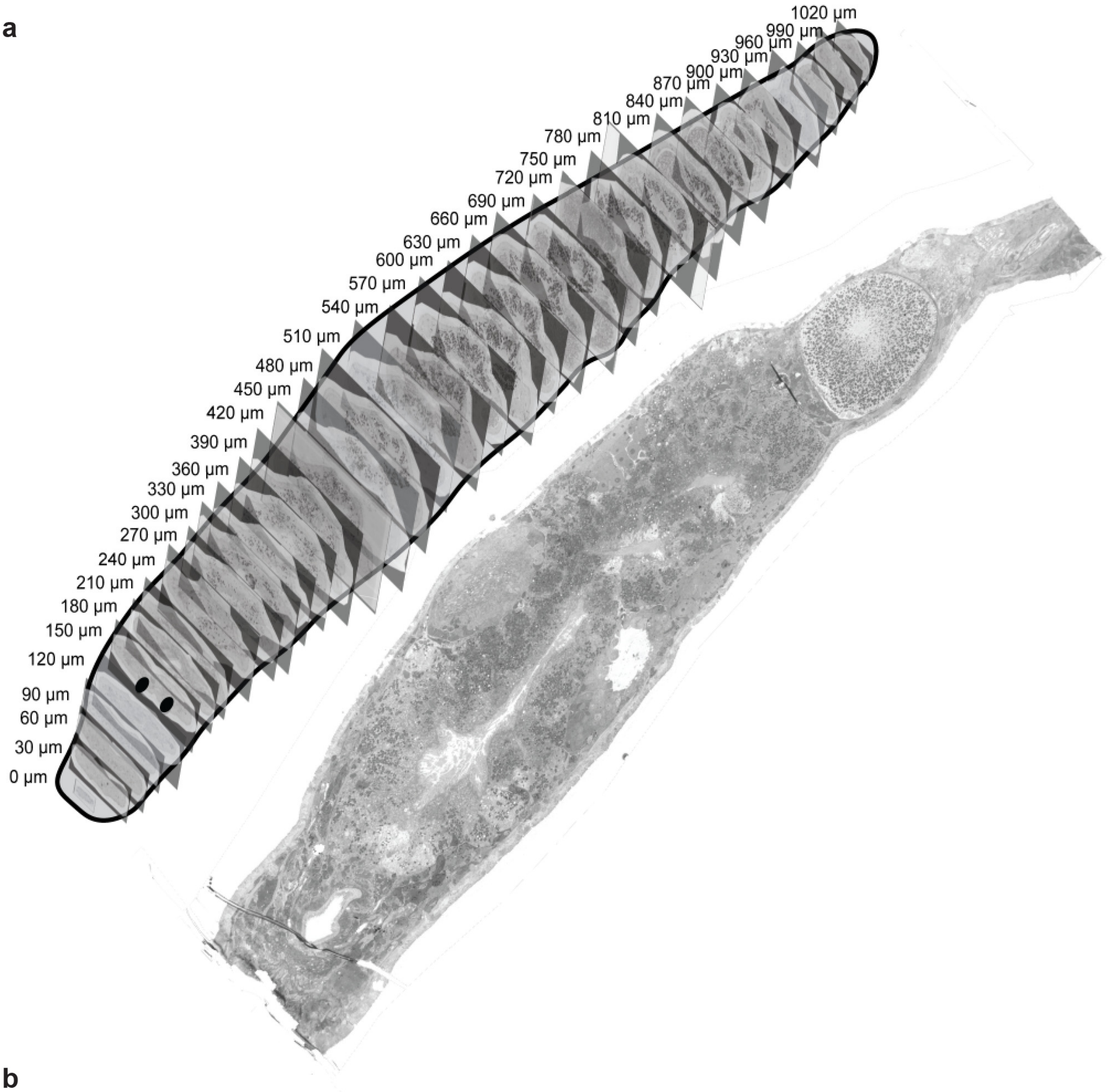

b

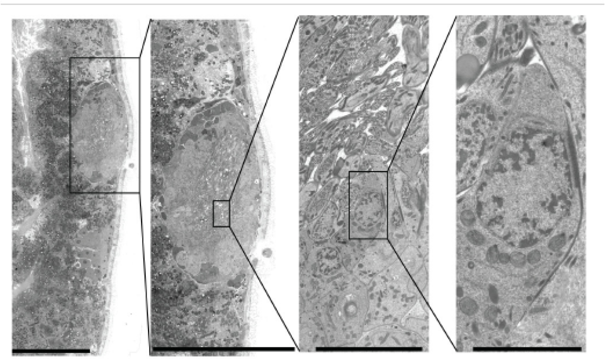

Supplementary Figure 2. Nanotomy in *M. lignano*. (**a**) One longitudinal section and 35 cross-sections through the entire adult animal with the 30 µm intervals were made. (**b**) An illustration of different magnification levels. Scale bars are 100 µm, 100 µm, 10 µm, and 3 µm. The data are accessible at http://www.nanotomy.org/OA/Macrostomum

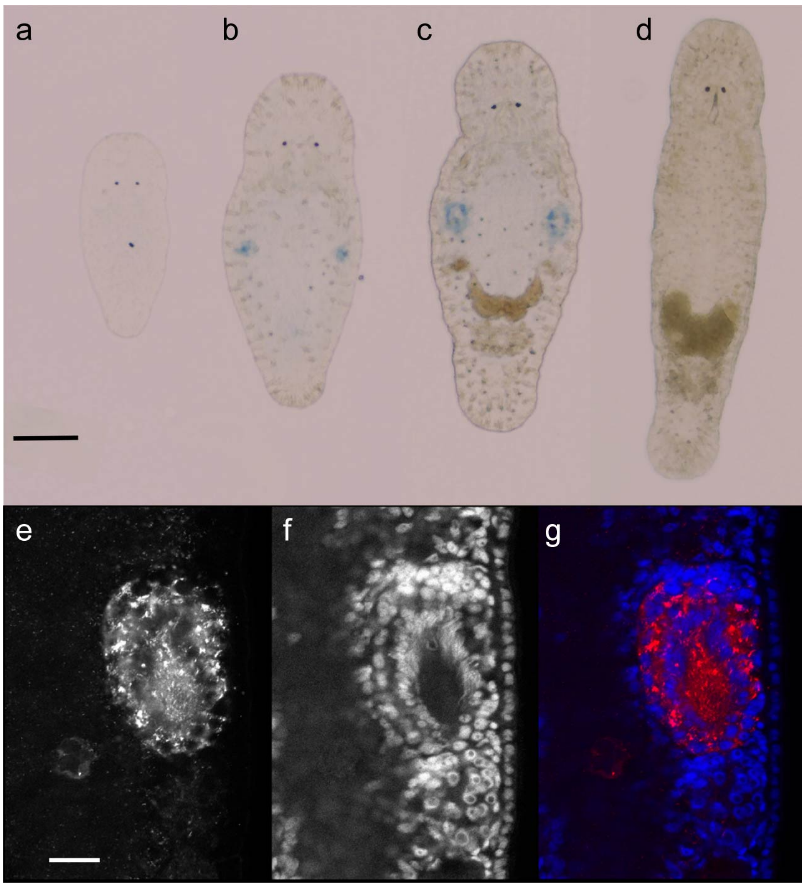

Supplementary Figure 3. Expression pattern of the *Mlig-sperm1* gene. (**a-d**) Whole mount *in situ* hybridization of *Mlig-sperm1* gene in a 1-day old hatchling (**a**), 4-days old juvenile (**b**), adult individual (**c**), and control WISH with sense *Mlig-sperm1* probe (**d**). (**e-g**) *Mlig-sperm1* FISH (**e**), with DAPI counterstain (**f**), and their overlay (**g**). This indicates the specific expression of *Mlig-sperm1* (red) within the testes and the lack of expression in the ovaries, which is represented by the dense cluster of DAPI-labeled (blue) nuclei under the testes. Scale bar: 100 μm (**a-d**) and 25 μm (**e-g**).

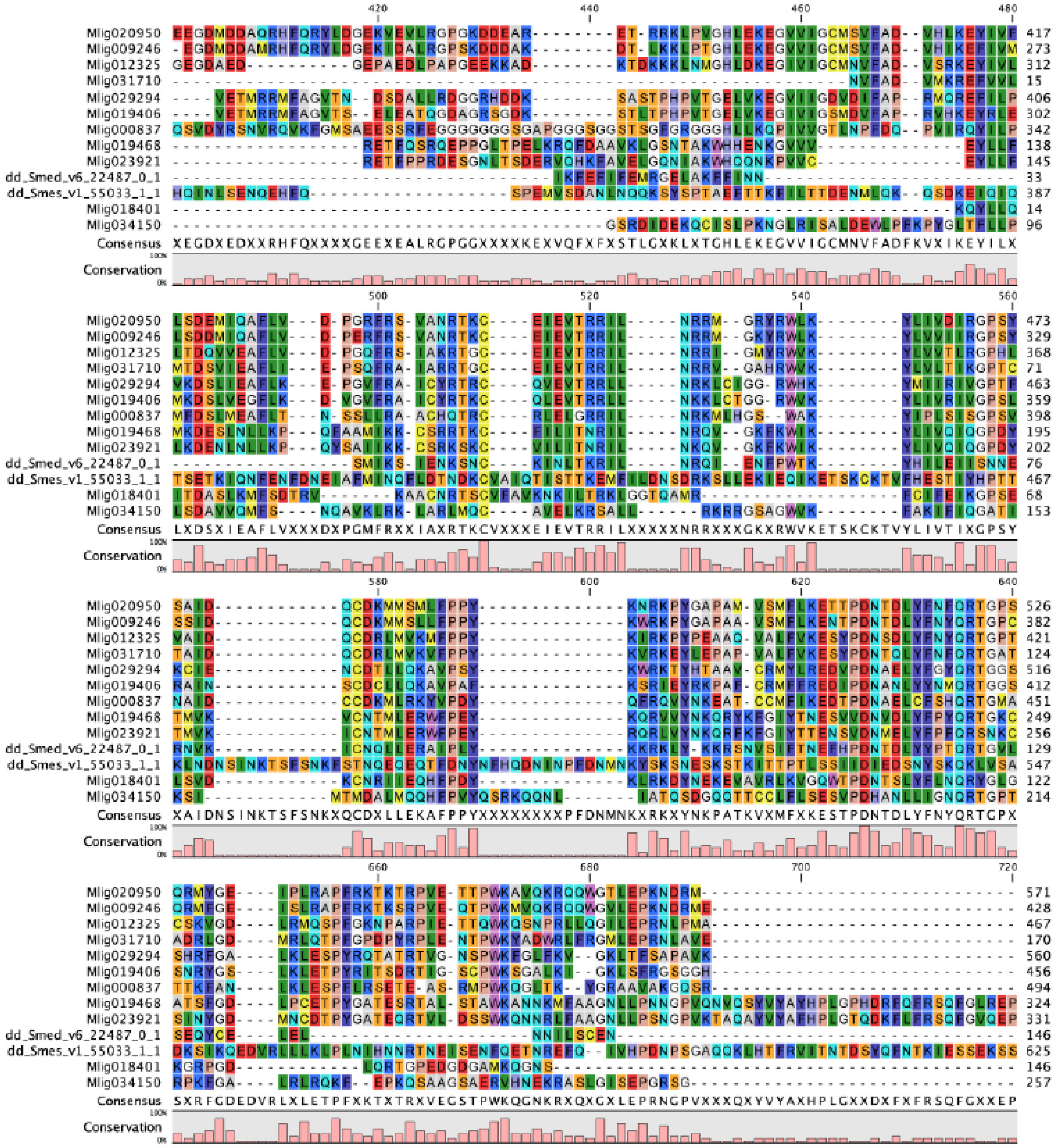

Supplementary Figure 4. Amino-acid alignment of representative members of the Mlig-SPERM1 gene family. Only the most conserved part of the alignment is show.

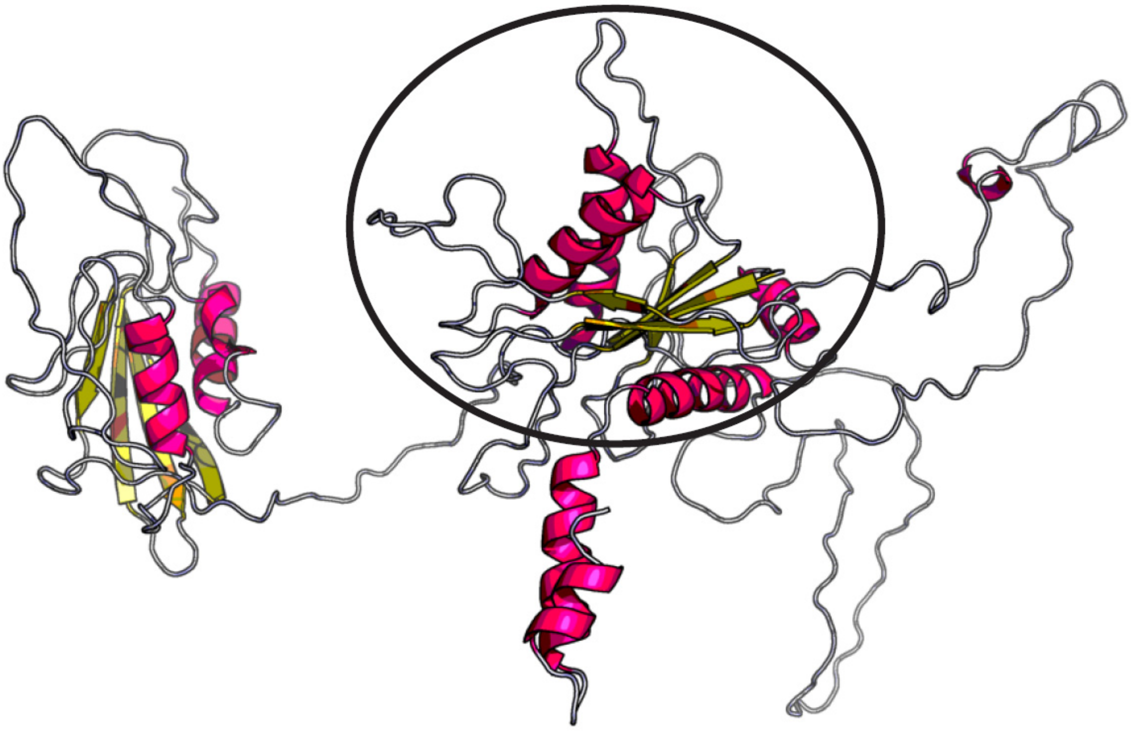

Supplementary Figure 5. Predicted tertiary structure of Mlig-SPERM1 protein. The conserved region of the protein is encircled.
